# Case Report: Two infants with hypercalcemia of rare and distinct etiologies

**DOI:** 10.3389/fendo.2026.1799236

**Published:** 2026-03-30

**Authors:** Ivona Butorac Ahel, Vereniki Andritsos, Đurđica Košuljandić, Srećko Severinski, Maja Ješić, Kristina Lovrinović Grozdanić, Kristina Baraba Dekanić, Jurica Jambrović, Iva Bilić Čače

**Affiliations:** 1 Department of Pediatrics, Clinical Hospital Centre Rijeka, Rijeka, Croatia; 2 Faculty of Medicine, University of Rijeka, Rijeka, Croatia

**Keywords:** CYP24A1 gene mutation, hypercalcemia, infant, nephrocalcinosis, pyuria, Williams syndrome

## Abstract

**Objectives:**

Hypercalcemia in infancy is rare and often presents with nonspecific clinical signs, making early recognition challenging. This report describes two infants with severe symptomatic hypercalcemia due to distinct etiologies and highlights the diagnostic value of sterile pyuria as an early clinical clue.

**Case presentation:**

Both infants presented with nonspecific symptoms, including poor feeding, irritability, constipation, and sterile pyuria. In the first infant the diagnosis of Williams-Beuren syndrome had been established earlier, while the second infant had been previously healthy. Both had demonstrated sonographic evidence of nephrocalcinosis. Laboratory evaluation revealed markedly elevated serum calcium levels (up to 4.73 mmol/L), hypercalciuria, and suppressed PTH concentrations in both cases. Standard treatment of hypercalcemia was ineffective, while a single dose of zoledronic acid led to normalization of calcium levels and complete resolution of symptoms. Subsequent genetic testing in the second infant identified a biallelic pathogenic variant in the *CYP24A1* gene, confirming the diagnosis of idiopathic infantile hypercalcemia type 1. During follow-up, neither patient experienced recurrence of hypercalcemia, and renal ultrasound showed improvement or stabilization of nephrocalcinosis.

**Conclusion:**

Sterile pyuria accompanied by nonspecific symptoms may be an early indicator of hypercalcemia in infants and should prompt calcium evaluation. To our knowledge, this is the first report linking sterile pyuria directly to hypercalcemia in this age group. Zoledronic acid can effectively normalize calcium levels in refractory pediatric hypercalcemia. This report adds to the limited evidence regarding zoledronic acid use in infants with Williams–Beuren syndrome and idiopathic infantile hypercalcemia type 1.

## 1 Introduction

Hypercalcemia is defined as a serum calcium concentration exceeding the upper limit of age-specific reference ranges (1). Accurate interpretation requires consideration of age-appropriate values, as calcium levels are physiologically higher in newborns and preterm infants and gradually decline with age. Hypercalcemia in infancy is a rare but clinically important finding, often indicating an underlying genetic, endocrine, or metabolic disorder. It is considerably less common in children than in adults, in whom primary hyperparathyroidism and malignancy predominate. The neonatal period and early infancy represent the age groups most commonly affected by hereditary and syndromic forms of hypercalcemia. Infantile idiopathic hypercalcemia (IIH) is a rare disorder, with prevalence varying between populations depending on mutation frequency. In contrast, hypercalcemia has been reported in 5–50% of children with Williams–Beuren syndrome (WS), typically presenting during the first two years of life (2).

While hypercalcemia in childhood is typically mild and asymptomatic, symptomatic cases in infants may present with nonspecific signs such as vomiting, constipation, abdominal pain, poor feeding, hypotonia, failure to thrive, polyuria, and polydipsia (3). Despite its subtle presentation, unrecognized or untreated hypercalcemia can result in serious complications, including nephrocalcinosis, neurodevelopmental delay, and decreased bone density. Prompt recognition and systematic evaluation are therefore essential to determine the etiology and prevent irreversible organ damage.

Here, we describe two infants with symptomatic hypercalcemia due to distinct and rare etiologies, emphasizing the diagnostic challenges and clinical variability of this condition.

## 2 Case presentation

### 2.1 Patient 1

#### 2.1.1 General information

A 10-month-old male infant with a known diagnosis of WS was admitted for evaluation of severe hypercalcemia. He was the first child of healthy, non-consanguineous parents and was born at term following an uncomplicated pregnancy. Birth weight was 2.840 kg (12th percentile), length 48 cm (16th percentile), and Apgar score 10/10. Dysmorphic features, a cardiac murmur and right-sided inguinoscrotal hernia were noted at birth. Transthoracic echocardiography revealed supravalvular aortic stenosis, and the diagnosis of WS was confirmed by genetic testing at two months of age. He was enrolled in regular multidisciplinary follow-up. At four months of age, routine nephrological follow-up had shown right-sided pyelectasis and hypercalciuria with normal serum calcium levels. Since the first month of life, he had received routine vitamin D supplementation (400 IU/day).

#### 2.1.2 Clinical findings

Approximately one month prior to admission, the infant became irritable and intermittently agitated. Parents reported occasional post-prandial vomiting after milk feeds, refusal of solid foods, and constipation. There was no fever. On admission, he weighed 7.750 kg (71st percentile), measured 73 cm in length (71st percentile), and had a head circumference of 46.5 cm (95th percentile). He appeared irritable and clinically dehydrated.

#### 2.1.3 Diagnostic approach

Approximately six weeks prior to admission, the infant was repeatedly treated with antibiotics for presumed afebrile urinary tract infections; however, urine cultures were sterile. Subsequent renal ultrasonography demonstrated bilaterally increased echogenicity of the renal pyramids, consistent with early nephrocalcinosis. Laboratory evaluation at admission revealed severe hypercalcemia (total calcium 4.27 mmol/L), hypercalciuria, suppressed PTH levels and normal serum 25-hydroxyvitamin D concentrations (**Table 1**). The findings were consistent with PTH-independent hypercalcemia most likely related to WS. Alternative causes of sterile pyuria, including urinary tract infection and structural abnormalities, were excluded. Although total calcium intake was not formally assessed, feeding was balanced and age-appropriate.

**Table 1:** Clinical features and laboratory findings of the two patients.

Patient's Data		Patient 1	Patient 2	Reference range
Sex		M	M	
Age at diagnosis		10 mo	4 mo	
Symptoms	Vomiting	+	+	
	Constipation	+	+	
	Poor feeding	+	+	
	Irritability	+	+	
	Urinary tract infection	–	–	
Laboratory results				Reference range
On the day of admission				
Total calcium (mmol/L)		4.27	4.73	2.15-2.80
Total magnesium (mmol/L)		0.95	1	0.65-1.03
Inorganic phosphate (mmol/L)		1.32	1.38	1.15-2.15
Urea (mmol/L)		5.7	5.2	1.0-7.5
Creatinine (μmol/L)		37	39	14-34
Uric acid (μmol/L)		318	389	65-330
Cystatin C (mg/L)		1.46	1.77	0.53-0.95
Beta 2 microglobulin (mg/L)		2.5	ND	1.09-2.53
PTH (pmol/L)		0.64	0.35	1.6-6.9
Urine Ca/Cr ratio (mmol/mmol)		4.08	3.08	0.09-2.20
Urine uric acid/creatinine ratio (mmol/mmol)		1.01	ND	0.70-1.5
Urine albumin (mg/L)		40	ND	< 20
Urine alb/Cr ratio (mg/mmol)		46.6	ND	< 5.0
25-OH Vitamin D (nmol/L)			> 385.5	> 75
Alkaline phosphatase (U/L)			139	25-100
On the day of discharge				Reference range
Total calcium (mmol/L)		2.58	2.46	2.15-2.80
Total magnesium (mmol/L)		0.76	0.8	0.65-1.03
Inorganic phosphate (mmol/L)		1.28	0.84	1.15-2.15
Urea (mmol/L)		3.5	6.3	1.0-7.5
Creatinine (μmol/L)		28	30	14-34
Alkaline phosphatase (U/L)		255	89	25-100
Urine creatinine (μmol/L)			604.8	
At last observation				Reference range
Total calcium (mmol/L)		2.54	2.71	2.15-2.80
Total magnesium (mmol/L)		0.79	0.9	0.65-1.03
Inorganic phosphate (mmol/L)		1.46	1.18	1.15-2.15
Urea (mmol/L)		3.5	1.9	1.0-7.5
Creatinine (μmol/L)		23	16	14-34
Imaging examination				
Nephrocalcinosis		+	+	

ND, not done.

#### 2.1.4 Therapeutic intervention

**Figure 1:**
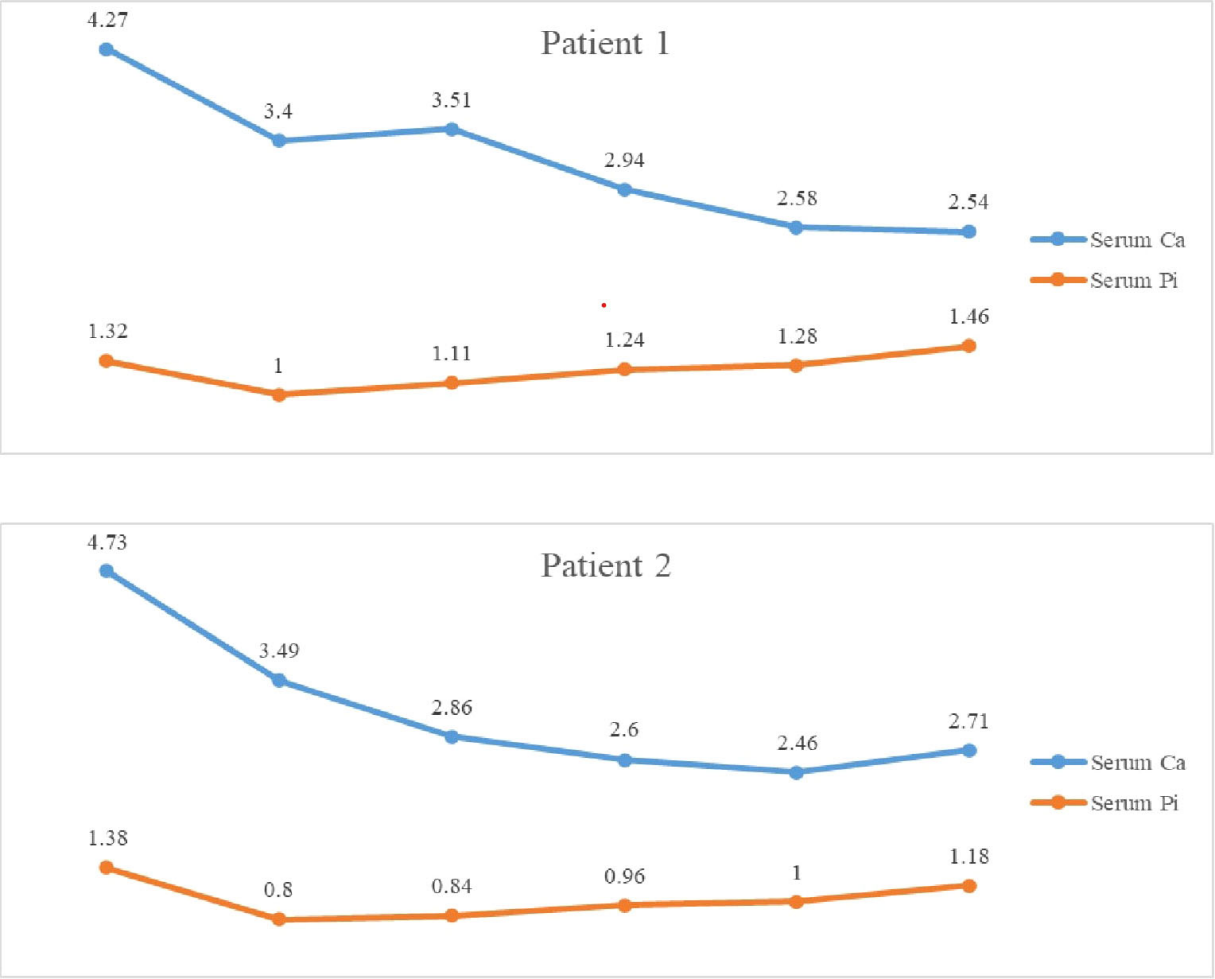
Trends in serum calcium and phosphorus levels throughout hospitalization. The numbers shown on the line graphs correspond to measured concentrations (mmol/L).

The treatment of hypercalcemia was initiated with hyperhydration (3 L/m^2^) and forced diuresis. In addition, vitamin D intake was discontinued and oral calcium intake was limited. Although calcium levels initially declined, they rebounded after 48 hours. Due to persistent severe hypercalcemia, a single intravenous dose of zoledronic acid was administered, resulting in normalization of serum calcium within 24 hours (**Figure 1**).

#### 2.1.5 Follow-up

The infant’s clinical condition and appetite improved promptly after normalization of calcium levels. He was discharged with instructions to avoid vitamin D supplementation and to use a modified low-calcium formula. During four years of follow-up, serum calcium levels remained within the normal range, the urinary calcium/creatinine ratio normalized, and renal ultrasonography showed improvement of nephrocalcinosis.

### 2.2 Patient 2

#### 2.2.1 General information

A 4-month-old male infant presented with failure to thrive and poor feeding. He was born at term after an uncomplicated pregnancy to healthy, non-consanguineous parents. At birth, his weight was 3.170 kg (76th percentile), his length 51 cm (72nd percentile), and Apgar score 10/10. He was exclusively breastfed until 3 months of age, when a milk formula supplementation was introduced. He had received routine vitamin D supplementation (400 IU/day) since one month of age.

#### 2.2.2 Clinical findings

At presentation, parents reported poor feeding and stools once weekly. There was no history of vomiting, fever, or apparent infection. By the time of hospital evaluation, he had lost 490 g compared to prior documented weight. On admission, he weighed 5.490 kg (2nd percentile) and measured 64 cm in length (50th percentile). Vital signs were normal. Moderate hypotonia was observed, without dysmorphic features or signs of systemic illness.

#### 2.2.3 Diagnostic approach

Urinalysis showed pyuria. Antibiotic therapy was started for presumed urinary tract infection; however, urine cultures remained negative. A week later, he was examined by a nephrologist who noted sonographic signs of nephrocalcinosis. Laboratory evaluation confirmed severe hypercalcemia (4.73 mmol/L), hypercalciuria, suppressed PTH levels and markedly elevated vitamin D concentrations (**Table 1**).

Excessive vitamin D intake was explicitly denied by the mother. Differential diagnosis included vitamin D intoxication, granulomatous disease, and disorders of vitamin D metabolism. In the absence of clinical or laboratory evidence of alternative causes, IIH was suspected. Subsequent genetic testing identified a biallelic pathogenic variant in the *CYP24A1* gene, confirming the diagnosis of IIH type 1. The same pathogenic variant was also identified in the older brother, who was clinically asymptomatic. The parents did not provide consent for their genetic testing.

#### 2.2.4 Therapeutic intervention

The treatment of hypercalcemia was initiated with hyperhydration (3 L/m^2^) and forced diuresis using intravenous furosemide (1 mg/kg), in combination with corticosteroids. Despite this therapy, calcium levels remained elevated, necessitating the administration of zoledronic acid. Additionally, the infant was fed with specialized low-calcium, vitamin D-free formula. A single intravenous dose of zoledronic acid (0.025 mg/kg) resulted in rapid normalization of calcium levels (**Figure 1**).

#### 2.2.5 Follow-up

The patient was discharged on day 8 with instructions to restrict dietary calcium intake and to strictly avoid vitamin D supplementation and excessive sunlight exposure. Throughout the two-year follow-up period, no recurrence of hypercalcemia or hypercalciuria were observed, and renal nephrocalcinosis showed no change on ultrasonographic evaluation.

Written informed consent was obtained from the parents of both patients for publication of this case report, and the study was approved by the Ethics Committee of the Clinical Hospital Center Rijeka.

## 3 Discussion

Hypercalcemia in infancy presents with a wide variety of nonspecific clinical signs and symptoms. Being significantly less prevalent in children than in adults, it may easily go unrecognized, as serum calcium is not routinely measured in pediatric biochemical panels (4). Consequently, the recognition of hypercalcemia in infancy can be a significant clinical challenge. When detected, it can indicate a serious underlying systemic, genetic, or metabolic disorder and warrants prompt investigation to prevent long-term complications (2).

The severity of hypercalcemia is best determined by measuring ionized calcium. When this measurement is unavailable, total serum calcium corrected for albumin can serve as an acceptable alternative. The diagnostic workup should begin with serum PTH measurement, followed by evaluation of vitamin D metabolites to help distinguish between PTH-dependent and PTH-independent causes. In neonates, infants and young children genetic causes are more likely to be the culprits of hypercalcemia, whereas in older children and adolescents acquired causes that are typically seen in adults are more prevalent (3). The etiologies of PTH-dependent hypercalcemia in infants include several isolated or syndromic disorders such as familial hypocalciuric hypercalcemia type 1 (FHH1), neonatal severe hyperparathyroidism (NSHPT) and secondary hyperparathyroidism due to maternal hypocalcemia or hypoparathyroidism. In infants with hypercalcemia and low PTH, nutritional causes, IIH, Jansen’s metaphyseal chondrodysplasia, WS, inborn errors of metabolism and subcutaneous fat necrosis should be considered as potential causes.

Both of our patients presented with hypercalcemia and suppressed PTH levels, consistent with PTH-independent hypercalcemia. In the first case, hypercalcemia occurred in the context of WS, a rare genetic disorder known to be associated with calcium metabolism abnormalities. Proposed mechanisms include increased intestinal calcium absorption, possibly mediated by enhanced sensitivity to active vitamin D metabolites, as well as altered renal calcium handling with reduced urinary calcium excretion. Hypersensitivity of the vitamin D receptor has been suggested as a contributing factor, amplifying the effects of circulating vitamin D metabolites on calcium absorption and bone resorption. Although these mechanisms likely contribute to the PTH-independent hypercalcemia observed in WS, the precise pathophysiology remains incompletely understood (5, 6). In the second case, the combination of extremely elevated vitamin D levels and suppressed PTH initially suggested excessive vitamin D intake. After excluding this possibility, as well as dysmorphic features and granulomatous disease, a defect in vitamin D metabolism was considered. In the absence of access to comprehensive metabolite profiling, a presumptive diagnosis of IIH type I was made, which was subsequently confirmed by identification of a biallelic pathogenic variant in the *CYP24A1* gene. IIH is a rare genetic disorder caused by mutations in the *CYP24A1* or *SLC34A1* genes, which encode key enzymes involved in vitamin D metabolism. Both mutations result in elevated levels of active vitamin D metabolites, persistent hypercalcemia, hypercalciuria and suppressed PTH levels. These typical biochemical abnormalities promote abnormal renal calcium depositions, increasing the risks of nephrolithiasis and nephrocalcinosis. Carriers of *SLC34A1* mutation additionally present with hypophosphatemia and renal phosphate wasting. Although termed “infantile”, the disorder is not limited only to infancy and may manifest later in childhood and adulthood (4).

Typically, in patients with *CYP24A1* mutation, vitamin D levels are mildly elevated or within the high-normal range. In cases where patients present with markedly elevated 25-hydroxyvitamin D (25-OH-D) concentrations, as in our case, excessive supplementation, dosing errors, or intake of fortified formula should first be considered. Additionally, analytical interference must be considered, particularly when 25-OH-D is measured using automated immunoassays, which may be susceptible to assay-specific limitations or cross-reactivity. Confirmation using liquid chromatography–tandem mass spectrometry (LC-MS/MS) and, where available, comprehensive vitamin D metabolite profiling (including 24,25-dihydroxyvitamin D and calculation of the 25-OH-D/24,25-(OH)_2_D ratio) can help distinguish true vitamin D intoxication from impaired vitamin D catabolism due to *CYP24A1* mutations (7, 8). In our patient, serum vitamin D levels were measured using an automated immunoassay, as more precise quantification methods were not available. Measurement of 24,25-dihydroxyvitamin D and calculation of the 25-OH-D/24,25-(OH)_2_D ratio could not be performed. The patient’s mother vigorously denied any excessive intake of vitamin D or other supplements, supporting the interpretation that the elevated 25-OH-D level reflects impaired metabolism rather than exogenous over-supplementation.

In both of our cases, sterile pyuria served as an important clue leading to further evaluation and ultimately to the diagnosis of hypercalcemia. Severe hypercalcemia results in hypercalciuria, which may lead to intratubular calcium crystal deposition and nephrocalcinosis. Crystal-induced tubular irritation and localized inflammatory response may manifest as leukocyturia in the absence of bacterial infection. In this context, sterile pyuria is likely reflected calcium-mediated tubular injury rather than a primary infectious process (9–11). In both infants, urine cultures remained repeatedly negative and no clinical or laboratory evidence suggested systemic inflammatory or granulomatous disease. The persistence of sterile pyuria triggered renal imaging, identification of nephrocalcinosis, and subsequent metabolic evaluation. Accordingly, it represented a clinically meaningful finding rather than an incidental observation.

Nephrocalcinosis and/or nephrolithiasis are well-documented complications of IIH, occurring in almost all affected individuals (12). In contrast, nephrocalcinosis is considered an uncommon finding in WS, occurring in only 5% of affected individuals (13). Although nephrocalcinosis increases the risk of developing chronic kidney disease, a study by Janiec et al. found no direct relationship between the severity of nephrocalcinosis and progression to end-stage renal disease (12).

In clinical practice, investigation of hypercalcemia often occurs concurrently with its management. Initial treatment focuses on correcting dehydration with isotonic saline, administering furosemide, reducing calcium and vitamin D intake, and targeting the underlying cause. In cases of refractory hypercalcemia that do not respond to standard therapy, bisphosphonates are administered. Pamidronate is most commonly used, acting by reducing osteoclastic activity (13). However, evidence on the use of zoledronic acid in childhood is scarce. In both of our patients, zoledronic acid was effectively used early in the management of hypercalcemia. This decision was based on the markedly elevated serum calcium levels at presentation which remained persistently high despite vitamin D withdrawal, dietary calcium restriction, and unsuccessful intensive intravenous hydration combined with furosemide therapy. Owing to the insufficient biochemical response and the unavailability of alternative bisphosphonate formulations, treatment with zoledronic acid was initiated. According to the available literature, bisphosphonates have been administered to only five reported pediatric patients with WS to date, and zoledronic acid use has been documented in a single case of IIH (14). After successful treatment of acute severe hypercalcemia and normalization of serum calcium concentrations, long-term management in both patients consisted of non-pharmacological strategies, including dietary calcium restriction, avoidance of vitamin D supplementation, and reduced sunlight exposure. Thus far, this approach has been effective in maintaining normocalcemia.

Long-term management of hypercalcemia in patients with WS involves regular monitoring of serum calcium, urinary calcium excretion, renal function, and blood pressure, as well as multidisciplinary follow-up due to the multisystem nature of the disorder. In contrast, long-term management of IIH requires lifelong vigilance regarding dietary calcium and vitamin D intake. In patients with *SLC34A1* mutations, therapy should additionally address phosphate deficiency and normalization of mineral metabolism. Rifampin has been proposed as a potential therapeutic option, as it may enhance alternative pathways of vitamin D metabolite inactivation; however, clinical experience remains limited (15, 16).

## 4 Parents perspective

The parents reported significant distress during the diagnostic process, particularly due to the nonspecific nature of symptoms and repeated evaluations for presumed urinary tract infection. They expressed relief following identification of the underlying cause and improvement after treatment.

## 5 Conclusion

This report highlights the diagnostic and therapeutic complexities of severe PTH-independent hypercalcemia in infants. It underscores that sterile pyuria accompanied by nonspecific symptoms may serve as an early clinical clue to underlying disturbances in calcium homeostasis and should prompt evaluation of serum calcium levels. Additionally, our findings contribute to the limited pediatric evidence supporting the use of zoledronic acid in infants with Williams–Beuren syndrome and infantile idiopathic hypercalcemia type 1.

## Data Availability

The original contributions presented in the study are publicly available. This data can be found in the ClinVar repository (https://www.ncbi.nlm.nih.gov/clinvar/). The relevant variant records are publicly available at the following accession links: https://www.ncbi.nlm.nih.gov/clinvar/variation/631878/ and https://www.ncbi.nlm.nih.gov/clinvar/variation/29677/.

## References

[1] Goltzman D Feingold KR Ahmed SF Anawalt B Blackman MR Boyce A . Approach to Hypercalcemia. Dartmouth: MDText.com, Inc (2023).

[2] Gorvin CM . Genetic causes of neonatal and infantile hypercalcaemia. Pediatr Nephrol. (2022) 37:289–301. doi: 10.1007/s00467-021-05082-z, PMID: 33990852 PMC8816529

[3] Stokes VJ Nielsen MF Hannan FM Thakker RV . Hypercalcemic disorders in children. J Bone Miner Res. (2017) 32:2157–70. doi: 10.1002/jbmr.3296, PMID: 28914984 PMC5703166

[4] Lietman SA Germain-Lee EL Levine MA . Hypercalcemia in children and adolescents. Curr Opin Pediatr. (2010) 22:508–15. doi: 10.1097/MOP.0b013e32833b7c23, PMID: 20601885 PMC2967024

[5] Park E Kim SC . Williams syndrome presenting as infantile hypercalcemia with acute kidney injury: a case report. CEN Case Rep. (2025) 14:764–7. doi: 10.1007/s13730-025-01010-4, PMID: 40627324 PMC12457234

[6] Meng H Jia YX Yang HM Gao X Li CG Xin GY . A case report of Williams syndrome with main clinical manifestation of hypercalcemia and gastrointestinal bleeding as the main clinical manifestations, and with an accompanying literature review. Brain Behav. (2023) 13:e3131. doi: 10.1002/brb3.3131, PMID: 37337730 PMC10454276

[7] Kaufmann M Schlingmann KP Berezin L Molin A Sheftel J Vig M . Differential diagnosis of vitamin D–related hypercalcemia using serum vitamin D metabolite profiling. J Bone Miner Res. (2021) 36:1340–50. doi: 10.1002/jbmr.4306, PMID: 33856702

[8] Kaufmann M Morse N Molloy BJ Cooper DP Schlingmann KP Molin A . Improved screening test for idiopathic infantile hypercalcemia confirms residual levels of serum 24,25-(OH)2 D3 in affected patients. J Bone Miner Res. (2017) 32:1589–96. doi: 10.1002/jbmr.3135, PMID: 28304097

[9] Bendig DW . The differential diagnosis of sterile pyuria in pediatric patients: A review. Glob Pediatr Health. (2021) 8:2333794X21993712. doi: 10.1177/2333794X21993712, PMID: 34017902 PMC8114235

[10] Muthukumar T Afaneh C Ding D Tsapepas D Lubetzky M . Sterile pyuria in the modern era: clinical spectrum and diagnostic yield. Am J Med. (2024) 137:446–53. doi: 10.1016/j.amjmed.2024.01.011, PMID: 38307150

[11] Grewal US Suneja M . Clinical approach to sterile pyuria. Adv Chronic Kidney Dis. (2015) 22:110–4. doi: 10.1053/j.ackd.2014.12.001, PMID: 25704348 PMC4445132

[12] Janiec A Halat-Wolska P Obrycki Ł Ciara E Wójcik M Płudowski P . Long-term outcome of the survivors of infantile hypercalcaemia with CYP24A1 and SLC34A1 mutations. Nephrol Dial Transplant. (2021) 36:1484–92. doi: 10.1093/ndt/gfaa178, PMID: 33099630 PMC8311581

[13] Sanjad SA Aoun B Yammine H Bassyouni A Karam PE . Pamidronate rescue therapy for hypercalcemia in a child with williams syndrome. Front Endocrinol. (2018) 9:240. doi: 10.3389/fendo.2018.00240, PMID: 29867772 PMC5968380

[14] Zheng Z Wu Y Wu H Jin J Luo Y Cao S . Successful treatment of hypercalcemia in a Chinese patient with a novel homozygous mutation in the CYP24A1 gene using zoledronic acid: a case report. J Pediatr Endocrinol Metab. (2023) 36:886–9. doi: 10.1515/jpem-2023-0212, PMID: 37358380

[15] De Paolis E Scaglione GL De Bonis M Minucci A Capoluongo E . CYP24A1 and SLC34A1 genetic defects associated with idiopathic infantile hypercalcemia: from genotype to phenotype. Clin Chem Lab Med. (2019) 57:1650–67. doi: 10.1515/cclm-2018-1208, PMID: 31188746

[16] Lenherr-Taube N Furman M Assor E Thummel K Levine MA Sochett E . Rifampin monotherapy for children with idiopathic infantile hypercalcemia. J Steroid Biochem Mol Biol. (2023) 231:106301. doi: 10.1016/j.jsbmb.2023.106301, PMID: 36990163 PMC10441173

